# Foreign

**Published:** 1840-08-29

**Authors:** 


### FOREIGN.

*Cases in Legal Medicine, with Remarks.* By D. Skae, Esq., F. R. C. S., and Lecturer on Forsenic Medicine.

I. *Case of suffocation during a state of Intoxication.*—The following case, interesting in many respects, is particularly so in relation to medico-legal inquiries regarding sudden death occurring in suspicious circumstances.

I was called on the 18th of January, 1839, to examine the body of a man, supposed by his friends to have been poisoned, in consequence of the circumstances in which he died. He had been a mason, and of intemperate habits. On the morning of the day on which he died, he had breakfasted on beef-steaks, and afterwards dined on the same, in a house near the Fleshmarket. It farther appeared, from the relation of his friends, that he had sat in a low shop in High street the greater part of the day, drinking with the customers who came in; and that about five o'clock in the evening, by which time he was in a state of intoxication, he was seized with vomiting. He vomited once or twice, and during the act fell down apparently insensible. He was immediately hurried into a coach and conveyed home. It was supposed that he had been dead before he could be got into the coach.

Two days after death, the body was examined by Dr. J. Y. Simpson and myself. The body was that of a strong, muscular, and well-formed man, aged forty-five, and presented all the external appearance of robust health. The limbs were rigid, and there was considerable lividity on the posterior surface of the body. The face was rather pale than otherwise; and the eyes somewhat sunken. The tongue was in its natural position, and the mucous membrane of the mouth of the natural colour. A pretty large fragment of butcher-meat was found between the fauces. On turning out the *larynx* and *trachea*, several portions of food were seen projecting from the glottis. Upon a further examination, the larynx and trachea, and about two inches of both bronchial tubes were found to be filled with articles of food, chiefly fragments of butcher-meat, of considerable size, which had apparently been swallowed whole.

The posterior parts of the lungs contained a large quantity of blood. The ventricles of the heart were nearly empty; the left side greatly hypertrophied; and the auriculo-ventricular opening so large as to admit five fingers.

The mucous membrane of the posterior part of the cardiac, and of the stomach, was mottled with red points and minute extravasations of blood, but retained its natural transparency and thickness.

The brain, which was rather softer than usual, was also more vascular, and, on being sliced, presented numerous bloody points, appearances which were more distinctly marked in the cerebellum than in the cerebrum.

Death, in this case, must, I presume, be attributed to asphyxia, produced by the imperfectly vomited matters from the stomach having been carried into the air-passages, during one or more efforts to inspire.

It is highly probable that sudden death has often occurred in similar circumstances, especially during the insensibility produced by intoxication, and death been either attributed to other circumstances, or its cause has remained unknown, when an examination of the trachea would have solved the mystery; a reflection which strongly points out the necessity of examining the air-passages in every case of sudden death which becomes the subject of legal investigation.

Dr. Paris, in alluding to the possibility of such an event as that detailed, occurring during intoxication, or in a spasmodic paroxysm, mentions a case where death resulted from a similar cause, during an attack of epilepsy. It happened in St. James's Workhouse, and fell under the particular notice of Mr. Alcock. The patient was seized, after a hearty meal of pork, with an epileptic fit, during which he died; when, upon opening the trachea, it was found to contain a quantity of animal matter, resembling the pork upon which he had recently dined.*

One of the most remarkable circumstances in the case which I have detailed, was the great quantity of food which had entered the larynx, and the fact that it extended from the epiglottis, beyond the bifurcation of the trachea.

The most common cases of foreign bodies in the wind pipe are those in which, during the act of deglutition, or while playing with some article in the mouth, a sudden inspiration, caused by coughing, laughing, or speaking, carries a small body into the larynx along with the current of inspired air. Innumerable cases of this kind are upon record, where fragments of bone, teeth, almond shells, cherry or plumstones, beads, buttons, beans, &c., have entered the larynx, trachea, or right bronchus, and have caused death in various ways. In some cases death occurs within a short time, preceded by great difficulty of breathing, or convulsions and other indications of certain changes

* Paris and Fonblanque, Med. Jurisprudence, ii. 58.

produced in the brain by the dyspnœa and incessant coughing. In other cases, the fatal event has ensued at a very considerable distance of time, being caused by laryngitis, bronchitis, pneumonia, or other affections. In others, the foreign body has been coughed up, or removed by surgical operation.

In another class of cases, death is caused directly by suffocation, where, during the progress of deglutition, large morsels are attempted to be swallowed, and, by becoming impacted in the pharynx in such a manner as to lie on the epiglottis and keep it shut, cause immediate death. A case of this kind is recorded by Bartholin.* Gilbert, the poet, terminated his existence in a similar manner. Dr. A. T. Thomson relates the case of a boy who died suddenly from this cause, while attempting to bolt a large piece of beef.† Several cases of the same nature are given by Mr. Porter, in his valuable work,‡ where death occurred almost instantaneously. In one of these, the obstruction was caused by some rags of lint with which a deficiency in the palate had been stuffed, and which, getting loose and becoming entangled in the morsel the person was about to swallow, stopped immediately over the epiglottis, and thus kept it closely shut down. In this case an elastic switch had been passed into the stomach, immediately after the accident, without meeting with the obstruction. Mr. Porter points out the obscurity attending these cases, and the difficulty of removing the foreign body; and recommends the operation of tracheotomy as the means best adapted to the extreme urgency of the case, and most likely to prove successful. If artificial respiration must be had recourse to, it can be performed with much greater ease through the artificial opening; or, if the patient is able to respire, the first inspiration made by him through the opening, will, in all probability, enable him to expire with sufficient force to throw the foreign body into the mouth.

In such a case as that recorded in this paper, the circumstance of the event occurring when the individual had not been engaged in eating, or had nothing in his mouth at the time, the obscurity of the symptoms, and the suddenness of death, might leave the surgeon in darkness as to the cause of the accident until the body had been opened. If called sufficiently early, it is possible, however, that an accurate diagnosis might be formed from the circumstances under which the event occurred, the symptoms, the ineffectual efforts to respire, (the heart continuing to act,) and the discovery of vomited matters in the mouth. In these circumstances a sufficiently confident opinion might be formed to justify the operation of tracheotomy; but it may be questioned whether the operation would be of any avail in such a case as that recorded here, considering the great extent to which the air-passages were blocked up.

Cases such as this are, perhaps, of most interest in their medico-legal relations. They are almost certain to occur in circumstances calculated to excite a suspicion of poisoning. The individual has been drinking, perhaps in the company of persons of dissolute habits or suspicious character, and after, it may be, becoming nearly insensible, vomits, and dies suddenly. Such, in some measure, was the case in the instance here recorded, and suspicions of poisoning were entertained in consequence of these circumstances.

An interesting case of a similar nature occurred, more recently, at Newcastle, for the particulars of which I am indebted to Mr. Glover, Lecturer on Chemistry in the Newcastle Medical School. A private of the 98th Regiment, died suddenly in a house of bad fame in the Sandgate. The girl in whose company the man was at the time of his death, immediately fled and concealed herself, but was subsequently secured until after the inquest.

It appeared in evidence at the inquest, which sat on the two following days, that the soldier had been drinking, and was in a maudlin s ate at the time of his visit to the house in which he died. He had lain down on the top of a bed, where he smoked a pipe for about five minutes, and then put down his head with his hand under it. The girl, who had been sitting at the fire in the same room, rose and went to him in about a quarter of an hour, in order to waken him, but found him "cold and stiff." When a surgeon had been called, he found the heart still beating feebly; although the pulse could not be felt at the wrist, he attempted to bleed, but after a table-spoonful of blood had flowed, the action of the heart was found to have ceased.

The body was examined on the following day by Dr. Embleton and Mr. Glover. The face, shoulders, and upper part of the chest, were of a livid colour. The brain was healthy. The heart and large blood-vessels contained a large quantity of dark fluid blood. In the mouth a quantity of half-digested bread was found lying on the tongue. In the larynx and trachea was found a quantity of frothy mucus tinged with blood, in which were imbedded a number of translucent white particles, looking exactly like minute particles of bread. These particles were found to be much more numerous in the bronchi, some of which were completely blocked up by them. In the bronchi these particles were imbedded in a brown substance, having all the characters of chyme, being acid, and yielding a precipitate with nitrate of silver. The contents of the stomach very closely resembled the substances found in the air-passages.

From the whole circumstances of the case, it

* Cist. Med. p. 450.

† Lancet, 1836–37. ii. 213.

‡ Observations on the Surgical Pathology of the Larynx and Trachea, pp. 223–228.

can scarcely be doubted, that death took place from the half-vomited matters of the stomach being carried into the air-passages during inspiration.

I have not been able to find in the records of medicine any cases exactly similar to those here related. It appears to me not unlikely that in many cases of sudden death occurring during intoxication, the cause may have been overlooked from inattention to the possibility of such an event, and from a neglect to examine the air-passages; a reflection which adds additional force to the caution, so often insisted upon by all medico-legal authorities, of minuteness and accuracy in every *post-mortem* examination which may become the subject of judicial inquiry.

Two cases are mentioned by Bonet in some respects similar to the foregoing.* Investigating the cause of death in the carcase of a calf, he found the windpipe completely choked up with the remains of its food. In the trachea of a boy he found the air excluded in the same way by a quantity of semi-putrid food. He states that in both cases the epiglottis was too small to cover the superior opening of the larynx, and concludes from this and from the semiputrid condition of the substances found in the windpipe that they must have entered by degrees, and thus gradually choked up the passage; an opinion, the correctness of which may be questioned, when the sensibility of the parts, and the great irritation caused by foreign bodies, is considered. It appears more probable that death had occurred suddenly, from the entrance, at once, of the half-digested contents of the stomach into the trachea, by their being inhaled during vomiting.

II. *Case of Poisoning with Laudanum.*—The following case of accidental poisoning with laudanum is one of considerable interest, not only as regards the smallness of the dose, but in regard to the detection of the poison by chemical analysis.

A gentleman, aged fifty-six, of robust form, temperate and regular habits, had been subject for several years to slight chronic catarrh, and occasional pains in the bowels, for which he sometimes took a pennyworth (two drachms) of the *Tinctura Opii Camphorata.* A few days before the accident, he had complained of slight flushing of the face and soreness of the eyes, and on the evening of Sunday 14th July, 1839, having been somewhat harassed by his cough, he sent to a druggist in the neighbourhood for his accustomed quantity of paregoric. The bottle sent having been several times at the same shop for a similar supply, had on it a label bearing the druggist's name, and the words "London Paregoric Elixir." It was returned with the same label, and the contents were taken a few minutes before ten o'clock, the gentleman's wife remarking at the time that it was surely laudanum, to which he replied, "do you not know that there is laudanum in paregoric?" Some water-gruel was prepared for him, some of which he took soon after the draught. His wife went to bed about eleven o'clock, and almost immediately fell fast asleep. About three o'clock in the morning, as she supposed, she was awakened by his rising to take a drink of the water-gruel, the remains of which he gave her at her request. She again fell fast asleep, and awoke about four o'clock, when she found him breathing very hard, and remembering the remark made regarding the draught, she became anxious and attempted to waken him, but found that he was insensible. She then alarmed a neighbour, and after various ineffectual attempts to rouse him had been made, they sent for medical assistance. This was obtained after considerable loss of time. He was first seen about seven o'clock by Mr. Jaap, who gave an emetic, and tried several means in order to rouse the patient, but all of these proving ineffectual, he called in additional advice.

About twenty minutes before nine, when I first saw the patient, eleven hours after swallowing the draught, I found the pulse very small and rapid, the face rather pale, the pupils contracted, the breathing somewhat loud and slow. After learning the smallness of the dose, and the time that had elapsed, I did not then think it advisable to waste time using the stomach pump. The means upon which I chiefly relied were cold water, poured in a continued stream upon the head, or dashed on the face, and dragging the patient up and down between two men. Various other means were also tried; in particular, the nostrils were made to bleed; small doses of carbonate of ammonia, and a considerable quantity of a strong infusion of coffee were given at intervals.

I was at first inclined to hope that these means would prove successful. The patient evinced a considerable degree of sensibility, especially when the water was dashed on his face, looking up, and contracting his features into that peculiar expression commonly put on by a person in the full possession of his faculties when similarly treated; and at the same time putting up his hands before his face and under his chin, so as to catch the drops of water. On one occasion, at least, when thus roused, he answered "yes" to a question. Notwithstanding the vigorous use of these and other means, the pulse rapidly became so enfeebled as to be imperceptible at the wrist, and he was soon incapable of exercising any muscular power in standing, although supported on each side. A small quantity of brandy and water was then put into his mouth which ran out; and he was immediately got into bed, with the intention of trying further means, but the breathing almost instantly ceased.

The bottle out of which the draught had been taken, was at the time examined by Mr. Jaap,

* Sepulch. Anat. Lib. ii. Sec. ii. Obs. viii.

Mr. Macfarlane, surgeon, at that time one of the most distinguished students of the extra-academical school, by another intelligent medical student present, and by myself, and in the opinion of all, distinctly smelt of laudanum, no odour indicative of paregoric being perceptible. A drop or two which remained in the phial enabled Mr. Jaap and myself to taste it, and neither of us had any hesitation in pronouncing it to be laudanum. The phial was examined in the evening by Mr. Lawrie, Clerk Street, who at first thought the odour was slight, but on holding the phial for a few moments in his warm hand, appeared to be convinced from the odour, as also from the taste of the moisture which remained, that it had contained laudanum.

On the sides of the phial, there were observed a number of small points, one or two of which appeared to be fragments of cork, but the rest rather suggested the idea that the laudanum had been poured from an unfiltered solution. The cork of the phial was quite entire.

The bottles containing the paregoric and laudanum in the apothecary's shop from which the medicine had been obtained, were afterwards examined. I found the bottles standing side by side on the shelf, the one containing laudanum being nearly full, the other about half empty. Both tinctures were quite transparent; but on the upper part of the laudanum bottle, above the surface of the tincture, I observed a number of minute points adhering to the inner surface of the glass, such as were seen in the phial, but smaller.

*Inspection.*—The body was examined two days after death. The external appearance was that of a man enjoying excellent health. The form did not appear to indicate an apoplectic tendency. There were no appearances of putrefaction, and the lividity observed on the posterior surface of the body was inconsiderable. On removing the cranium, a considerable quantity of dark fluid blood was found in the sinuses of the *dura mater*. The larger veins on the surface of the cerebrum and cerebellum were turgid, but not to any remarkable extent. Over the whole surface of the hemispheres, serous effusion was found beneath the arachnoid. About a drachm and a half of fluid was contained in one lateral ventricle, and about half a drachm in the other. The substance of the brain was in every respect natural, and no where presented any morbid appearances.

Some old adhesions were found between the surfaces of the pleura. The lungs contained, posteriorly, a considerable quantity of blood, which flowed out mixed with frothy mucus, on sections being made; but they crepitated perfectly in every part, and presented no alteration of structure.

The heart was healthy, and its different cavities of their natural size; the left side was empty, but the right auricle and ventricle contained black semi-fluid blood and a fibrinous clot, which extended for some inches along the pulmonary artery.

The viscera of the abdomen presented no morbid appearances. The stomach contained what appeared to be the remains of some gruel, and a few dark points, evidently coffee grounds. The duodenum contained also a similar fluid. These fluids were collected, the inner surface of the stomach being carefully washed out. No odour of opium could be perceived.

The bladder contained six ounces and a half of urine, which was collected. There was no disease of the genital organs.

The quantity of urine collected induced me to analyse it, although without any hope of detecting in it indications of opium. It was accordingly examined in the manner adopted by M. Barruel, who has represented himself as successful in a search of this kind, but no trace of morphia could be discovered.

In examining the contents and washings of the stomach, I pursued the method recommended by Dr. Christison, in his valuable treatise. The steps adopted may be briefly enumerated. The fluid was acidulated with acetic acid, stirred and filtered. The filtered fluid was evaporated in a vapour-bath to the consistence of syrup, redissolved in strong alcohol, boiled, filtered, and again evaporated to the consistence of syrup; this was redissolved in distilled water and again filtered. A solution of acetate of lead was then added as long as precipitation took place, and the precipitate separated by filtration.

(*a.*) The precipitate, being washed and suspended in water, was treated with a stream of sulphuretted hydrogen. After filtration and boiling, the fluid was concentrated by evaporation. This, amounting to about ten minims, slightly coloured, struck a deep olive-brown, with a single drop of a solution of the permuriate of iron; very different from the cherry-red colour struck with permuriate of iron, in a pure solution of meconic acid, but somewhat similar to the colour given in a comparative experiment, in which the solution of meconic acid was slightly coloured with coffee.

(*b.*) The filtered fluid was also treated with sulphuretted hydrogen and filtered. Ammonia was then added as long as precipitation took place. The precipitate, after being collected, washed, and drained on a filter, was boiled in alcohol, and the alcoholic solution, being filtered, was evaporated to dryness. A crystalline residue was obtained, slightly coloured, a small portion of which, on being brought in contact with a drop of nitric acid, assumed an orange colour, and then passed into a bright yellow solution.

In order, if possible, to get rid of some of the colouring matter, the remaining part of the crystalline mass, obtained in the second part of the process, was redissolved in alcohol and digested in a little animal charcoal. It was then filtered and again evaporated. The mass now obtained, somewhat purer, gave a distinct orange colouration with nitric acid. A small quantity of it was also added to a mixture of iodic acid and starch, moistened and mixed together on a white porcelain vessel; one part of the mixture immediately presented a deep blue colour, and the rest was converted into a reddish brown.

The results of these experiments were seen also by Mr. Kemp, and he agreed with me in thinking that the colour produced by the permuriate of iron, in itself a fallacious test, afforded an indistinct indication of meconic acid; and that the changes produced by nitric acid, and by iodic acid and starch, supplied unequivocal evidence of the presence of morphia.

The history of the preceding case presents, at first sight, several remarkable features, at variance with the commonly received opinions as to the effects of opium, and the difficulties attending its detection in the contents of the stomach.

1. *Cause of death.*—Before the result of the chemical analysis was known, several opinions were entertained and propagated as to the cause of death.

It may be remarked, generally, that, from all the circumstances of the case, it cannot be doubted that the only medicine taken by the deceased before his death was a seidlitz powder, and the contents of the phial as obtained from the druggist, whatever they were; death, therefore, was either caused by this or by disease. The young man who sold the drug confidently denied that he could have given laudanum, and asserted that death must have resulted from disease. Many who appeared even to be satisfied that the odour of the phial afforded sufficient evidence that laudanum had been taken, yet contended that the individual had died of serous or simple apoplexy. This opinion they founded partly upon the previous history of the individual as affording some proof of a tendency to apoplectic disease; partly also upon the smallness of the dose, but chiefly upon the presumption that the patient having risen out of bed at three o'clock, five hours after taking the draught, was incompatible with the known effects of opium.

As to this opinion, it may be remarked, in the first place, that before any chemical analysis of the contents of the stomach had been made, the proof of laudanum having been taken, appeared to me to be complete. The smell of a substance so familiar and peculiar, distinctly recognised and attested by several persons, appears to me to be sufficient evidence of its nature, especially when it is corroborated by the taste of the liquid, and by the testimony, also, of the boy who bought the medicine, who accurately described the colour of the liquid, and stated that the bottle was almost full, which was the case with the laudanum bottle, while that containing the paregoric, as we afterwards found, was half empty.

2. The grounds upon which it was supposed that the deceased had been predisposed to apoplexy were, that his father had died of that disease, and that he himself had once, several years before his death, suddenly fallen down; and that he had occasionally complained of flushing of the face, where he had also had one or two slight erysipelatous attacks with soreness of the eyes. These are certainly but very doubtful signs of such a predisposition. The falling down appears to have been a syncope, for it was a momentary occurrence, being neither preceded nor followed by any uneasiness. Although the other complaints may, in the opinion of some individuals, indicate what is mysteriously and ignorantly called a determination of blood to the head, or rather to the face, it is exceedingly questionable whether they can be connected with a habit of body predisposed to apoplexy. As to the father having died of apoplexy, it cannot be doubted that the tendency to this disease is frequently transmitted from parent to son; but this is by no means invariable, and the existence of disease in the parent, therefore, can only be considered as affording corroborative evidence of the existence of a predisposition in the son, when that is rendered probable by other circumstances. But granting that such a predisposition existed, it is surely unphilosophical to resort to remote and problematical causes, if the one known to be in operation be sufficient to account for the phenomena. 3. Was then the quantity of laudanum taken in this case, (two drachms,) sufficient to cause death? I think it cannot be doubted that it was; for, although this quantity may have been given with no injurious effects to persons suffering under violent pain or excitement, yet the known effects of two or three grains of opium, given medicinally to procure sleep, may readily lead us to believe that a quantity of laudanum equal to ten grains, would cause death. In this opinion I am supported by that of Dr. Paris, who says that four grains may cause death;* and by that also, I conceive, of Professor Christison, who relates, as confirmatory of the statement of Dr. Paris, a case which occurred to Dr. W. Brown, of this city, where a dose of four grains and a half of opium, taken by an adult, along with nine grains of camphor, was followed by the usual signs of narcotism, and death in nine hours.† Little of that insensibility to the effects of opium, produced by habit, could be supposed to exist in the case under consideration, for, although the person had been accus-

* Paris and Fonblanque's Medical Jurisprudence, ii. 388.

† Professor A. T. Thomson states that half an ounce of the tincture has proved fatal.—Lancet, 1836-7, ii. 189.

tomed, when affected with cough or pain, to take two drachms of paregoric, yet this quantity, which contains only the soluble part of half a grain of opium, was only taken at considerable intervals of time, and was never exceeded; and it does not appear that insensibility to the effects of a large dose of any narcotic is produced by the frequent use of a very small dose, but only when the dose is gradually increased in size. In addition, it will immediately be seen, as indeed might be suspected from the detection of the poison after death, that the quantity of laudanum taken was probably somewhat more than was at first supposed. 4. As to the circumstance upon which the opinion, that apoplexy was the cause of death, chiefly rested, namely, that the individual had spontaneously risen out of bed and taken a drink, five hours after having swallowed the draught, the importance of this fact, in determining to what cause death should be attributed, and the singularity of it, if death were really caused by poisoning, induced me to make very careful inquiries into the circumstances.

On questioning the widow at different times as to the time at which her husband rose, I learnt that, in the first place, she had been sound asleep before he rose, and fell fast asleep immediately after, and that she only guessed it to be three o'clock, from the time which she supposed she had passed in sleeping. Again, on questioning her as to the degree of light, she first stated that it was quite dark, so that her husband had to grope for a drink, and then that there was sufficient light to enable her to see the figure of her husband between her and the window, from which she imagined that the day had begun to break. Now, at the time this accident happened, (the 14th of July,) it must have been perfectly light at three o'clock in the morning, and as the blind of the window only was drawn, the room would never have been described as quite dark had it been that hour; and again, there is at that season of the year, sufficient light, from sunset to sunrise, to enable one to distinguish the bars of a window through the blind, and light certainly sufficient to enable one to distinguish a figure moving between him and the window; and as this is all which was observed in this case, the window being directly opposite the bed, it is, I think, not improbable that it may have been one, twelve, or even half-past eleven when this event happened. Although these statements render it probable that the individual may have risen at any period after eleven o'clock, the time at which his wife fell asleep, still it must be regarded as a circumstance somewhat singular, that a person should have been capable of spontaneously rising at a period certainly considerably greater than one hour, after a dose of laudanum had been taken, which ultimately proved fatal.

In poisoning with opium, the stupor is in general completely formed in less than one hour, especially when it has been taken in solution; yet there are not wanting cases in which its formation has been delayed for a longer period. Such a case is related in Corvisart's Journal, where two ounces and a half of the tincture, with a drachm of the extract, were swallowed, and no material stupor followed, until considerably more than an hour had elapsed.* A case is related by Mr. Shearman, which shows that certain circumstances may retard the operation of opium. In this case, intoxication produced that effect. A habitual drunkard, while intoxicated to excitement with beer and spirits, took two ounces of laudanum, and had no material stupor for five hours, during which period vomiting could not be induced, and he eventually died from the effects of the narcotic.† Professor A. T. Thomson refers to a case apparently of pure poisoning with opium, where an ounce of the tincture was taken at midnight; and in which the person slept and awoke at his usual hour, without having felt any of the influence of the poison. After rising, vomiting came on, and continued all day, but no other bad effect followed.‡ This was a case, certainly, which was not ultimately fatal; but, at the same time, it may be remarked, that the dose was a fatal one to a person unaccustomed to laudanum, and that the individual in this case was probably saved by the spontaneous occurrence of vomiting.

These cases are, I conceive, sufficient to show that such a variation from the usual effect of opium in poisonous doses is occasionally met with; and that this, in all probability, happened in the case under consideration. The length of time which elapsed in this case before the soporific effects of the poison were developed, may, perhaps, be attributed partly to the smallness of the dose, which was one little more than barely sufficient to cause death; and partly also to the circumstance of its being taken along with water-gruel, from which cause the absorption of the poison would go on more slowly. Another possible explanation suggests itself, namely, that the supervention of natural sleep may have retarded the absorption of the poison, or its action upon the nervous system. The occurrence of sleep certainly appears to retard the operation of the irritant poisons,§ but I am not aware of any facts which tend to show that it exerts a similar influence upon the agency of narcotics, unless the case here recorded, and the one cited from Dr. Thomson's lectures, can be regarded as affording some evidence in support of such an hypothesis.

In addition to the general character of the symptoms preceding death in this case, the paleness of the face, the quick pulse, and contracted pupils, &c., one circumstance will be

* Christison on Poisons, 648. † Ibidem.

‡ Lancet, 1836-37, ii. 789.

§ See Christison, op. cit.

observed in particular, which, I think, strongly confirms the opinion that death resulted from the operation of a narcotic poison. I refer to the circumstance that, even one hour, or an hour and a half before the death of the individual, he was still to a certain, although to a limited extent, capable of being roused, and displayed unequivocal signs of sensibility. This, so far as I am aware, has never been observed, at least, so short a time before death, in a case of apoplexy, fatal in twelve or thirteen hours.

*Lastly*, the morbid appearances taken in connection with the moral evidence of laudanum having been taken, and the character of the symptoms, and the rapidity of their progress to a fatal termination, can leave very little doubt that death was caused by opium; for although the morbid appearances were such as might have been met with in serous apoplexy, yet there is no case, I believe, upon record, of simple serous effusion to that extent having caused death in so short a space of time as twelve hours.

Such were the views of this case, and such the data upon which I ventured, previous to the completion of the chemical analysis of the contents of the stomach, to give a pretty positive opinion as to the cause of death. The subsequent detection of the poison affords unequivocal evidence that death resulted from its operation, for I believe it will be admitted to be in the highest degree improbable that morphia could be detected by its chemical tests after being twelve hours in the living body, without having been taken in sufficient quantity to cause death. I have chosen, in reviewing this case, to examine how far an opinion could be given from the symptoms and morbid appearances alone, as it is very unlikely that any evidence would have been obtained by chemical analysis, in a case where so small a quantity of opium had been taken. As the medical jurist has frequently failed to detect any traces of opium, in cases where a much larger quantity has been taken than in this instance, and where it had been retained, too, for a much shorter time in the stomach during the continuance of life, it is of the highest importance to know how far he can found an opinion as to the cause of death, on the character and progress of the symptoms, and on the morbid appearances alone; and it is interesting to find that, in a case like this, in several respects obscure and anomalous, at first sight, a pretty positive opinion could be given from these sources of evidence, which was afterwards fully corroborated by the detection of the poison.

2. *Detection of the Poison.*—Impressed with the fact, that the most experienced chemists have occasionally failed in obtaining any, or, at least, but faint indications of morphia and meconic acid, in analysing the contents of the stomach after quantities of laudanum, varying from half an ounce to two ounces, had been swallowed, and the fluid had remained but a few hours in the stomach, I undertook an analysis in this case, rather from a sense of duty than from any hope of obtaining evidence of the poison by its tests. Believing that only two drachms of laudanum had been taken, and that that quantity had caused death, I concluded that in all probability it must have been totally absorbed; and that it was therefore hopeless to look for any traces of it; the more so, as I was aware of the statement of Professor Christison, that only very faint indications could be obtained when the soluble part of ten grains of opium, a quantity at least equivalent to two drachms of laudanum, was actually put into an organic mixture, and that mixture examined by the same method as I pursued. I was therefore not a little surprised when I obtained what I consider to be distinct evidence of the presence of morphia, and was led immediately to suspect that a much larger quantity of laudanum than was at first supposed, had been taken. In consequence of this suspicion I carefully examined the boy who had been sent to purchase the medicine, and obtained from him so accurate and circumstantial an account of the manner in which the laudanum had been measured, as to leave no doubt in my mind that this was actually the case. After accurately telling the place where the laudanum bottle stood in the druggist's shop, and mentioning that it was nearly full, which was the case, while those next it were not so, he described how the laudanum had been measured, without any leading questions being put to him. From the description, it would appear that the young man had poured out a quantity of laudanum into the measure, and looked at the graduations on the glass, but after pouring it into the phial brought for the medicine, (an ounce phial,) he seemed to think that there was too much, for he returned a small quantity of it into the measure, and, without examining by the scale how much he had poured out, returned it into the original stock. On repeatedly putting the phial into the boy's hand, and asking him to point out with his finger how much liquid had been put into it, he invariably placed his finger on a point which indicated that the phial had been three-fourths full at the first measurement, and half full after the measurement had been corrected, and the phial given to him by the druggist. From the smartness of the boy, the circumstantial manner in which he detailed the facts, and his consistency in repeating them, as well as from the circumstance that it was impossible, almost, at his age, that he could have conjectured to what conclusions they would lead, I have no doubt that the phial had contained about half an ounce of laudanum.

Granting, then, that half an ounce of laudanum had been taken, the case must still be regarded as interesting, and shows the importance of pursuing the chemical analysis, even in cases where little hope can be entertained of deriving additional evidence from that source. I am not aware of any case upon record where so small a quantity of opium has been detected by chemical tests. There are, however, several cases to be found where the same quantity of laudanum had been taken, and where life had been prolonged nearly as long as in this instance, and where unequivocal evidence of another kind was obtained from the contents of the stomach after death. The following are cited by Professor Christison. "In Knape and Hecker's Register, there is the case of a girl who died about eight hours after taking half an ounce of laudanum; and the reporters found, that an extract prepared from the contents of the stomach caused deep sleep in frogs, chickens, and dogs, and threw some of them into a comatose state, which proved fatal. Wildberg has related a very interesting case of a young lady of Berlin, who had been seduced, and, finding herself pregnant, swallowed about half an ounce of laudanum in the evening, and died during the night. In this instance, the contents of the stomach had a narcotic odour, and their extract, when given to a young dog, caused excessive sleep, reeling, drunkenness, palsy of the legs, convulsions, and death. M. Petit has related another case, fatal in about ten hours, where the contents of the stomach had the smell of opium; and their alcoholic extract had a bitter taste, and killed guinea pigs with symptoms of narcotism."* In this instance it was estimated, that about a drachm of the extract had been taken.†

With reference to the case which is the subject of these remarks, I made several comparative experiments, in order to ascertain how small a quantity of laudanum in a mixed fluid would afford evidence of morphia and meconic acid. On adding successively to three mixtures of about three ounces of water-gruel each, with salt, two drachms, a drachm and a half, and one drachm of laudanum, I found that by careful manipulation, unequivocal evidence of the presence of opium was obtained from each.

The residue of the process for the detection of the morphia gave a red colour with nitric acid, passing gradually into a yellow solution. That for the detection of meconic acid assumed a deep reddish-brown with perchloride of iron. I found that in several instances in using iodic acid and starch, as a test of the presence of morphia in these experiments, that the brownish yellow colour, characteristic of the action of iodic acid on morphia, was produced, although no evidence of free iodine was obtained by its action with the starch. This might have been anticipated, considering the very minute quantity of morphia which must have been present. When a considerable excess of iodic acid is added to a minute quantity of morphia and starch in solution, the blue colour produced is always extremely evanescent, the whole solution rapidly assuming a permanently brownish yellow colour, in which no free iodine is indicated by starch: the blue colour cannot be recalled but by the addition of more morphia. This circumstance distinguishes the action of morphia with iodic acid, from that of albumen with the same substance, which (as pointed out by Mr. Davidson) also decomposes it, and sets the iodine free: in the latter case, the blue colour is more lasting, and when it ultimately disappears leaves the solution colourless. It is almost unnecessary to say that in the process pursued for the separation of morphia from mixed fluids, any albumen which may have been originally present will be entirely removed.

III. *Second Case of Poisoning with Laudanum.*—I was lately permitted, by the kindness of Professor Alison, and Dr. John Reid, to examine the body of a woman who had died in circumstances somewhat resembling those mentioned in the preceding case. The female referred to, had been under Dr. Alison's care in the Royal Infirmary, some weeks previous to the accident, and at that time had been affected with rheumatism. Two days before her death she had pain in her bowels, for which she had taken a pennyworth of laudanum. On the following evening she again sent for the same quantity, but appeared to have got considerably more, for the girl who bought it, remarked when she brought it home, that she had "gotten a muckle pennyworth," and that it was about twice as much as she had got the previous night. The character of her neighbours and friends prevented me from obtaining a very accurate account of matters, or one on which much reliance could be placed; but it appeared that the whole of the laudanum had been taken about ten o'clock: that about twelve or one a neighbour had looked in and asked her how she felt, and that she had replied cheerfully and readily. Her husband awoke about five, and spoke to her, but received no answer; he then fell asleep, and on again awakening, discovered that she was dead.

On examination of the body, no morbid appearances of any note were discovered in the viscera of the trunk. The external table of the left temporal bone was carious, and the temporal muscle at that part infiltrated with purulent matter. The arachnoid membrane was elevated and separated from the surface of the brain by serous fluid, and an inconsiderable quantity of serum was found in the right lateral ventricle.

A careful analysis of the contents of the stomach was made, but the only evidence obtained from this source was an imperfect and equivocal colouration on bringing the residue of the process for the separation of morphia into contact with nitric acid; and a faint bitter-

* Op. cit. p. 639.

† Journ. de Med. xxiv. 272.

ness on applying a small portion of it to the tongue.

This case in part corroborates the inferences which I have ventured to draw from the one which precedes it. It is to be regretted that a more circumstantial account of the symptoms preceding death could not be obtained. The absence of any morbid appearances sufficient to enable us to ascribe death to natural causes; the quantity of laudanum which had been taken; and the rapidity of the fatal termination, render it highly probable that the woman died from the effects of the poison. If this was the case, it would appear that narcotism had not been formed for several hours after the laudanum was taken.

To the foregoing cases many might be added, calculated to show the carelessness with which medicines are occasionally dispensed. A few simple regulations to be observed by the druggist in the arrangement of the more active medicines would prevent the occurrence of such a mistake as that related in the first of the foregoing cases. Nor is it, I believe, to be doubted, that fatal accidents might be prevented, and that much advantage would accrue both to physicians and patients in ordinary circumstances, were some means adopted to insure a uniformity in the strength and quality of the active medicines in common use. My own experience coincides, I believe, with that of all who have bestowed any attention on the subject, and bears me out in the conviction, that many patients in this place are every day receiving medicines which are nearly inert. The consequences which may result from such a circumstance are easily conceived. The physician is disappointed in the effects of his remedies, and his patient suffers misery from the delay. Or if, on the other hand, an individual has habituated himself to the use of some active drug, such as laudanum or prussic acid, and has gradually increased the dose of a preparation obtained at a particular shop, and should accidentally purchase his accustomed allowance at another shop, he may be poisoned by receiving a medicine which is double the strength of that which he has been accustomed to take.

*Ed. Med. and Surg. Journ.*

---

*Remarks on M. Roux's operation for Fistula in Ano.*—M. Roux, of the Hôtel-Dieu, Paris, performs this operation in a different manner to that performed by Pott, and followed by English surgeons. He introduces a long piece of boxwood into the rectum, having its concavity towards the fistula. A silver director is then introduced along the fistulous track, and its end made to come in contact with the wooden gorget in the bowel. A long, strong, narrow, sharp-pointed knife is then introduced along it, till it comes in contact with the piece of boxwood. The director is then withdrawn, and by keeping the point of the knife fixed upon the gorget, and withdrawing both together, all the parts between the fistula and the rectum are divided. This part of the operation completed, the bistoury is exchanged for a scalpel, and all the hardened base is carefully dissected out. A thick long probe is then procured, having a button at one end; this is covered with charpie, smeared over with some yellow-looking ointment, and the wound crammed full of it to the bottom.

This operation I ever have regarded as open to three objections. 1st. The boxwood gorget can only be of use in saving the finger of the surgeon, which is not very likely to be injured; and if the finger be not employed, the sense of touch cannot be available, and we are operating in the dark with nothing to guide us. Nor is it a very easy matter to introduce the point of a director at once through the opening into the rectum; this is admitted by M. Richerand, who adds, "that in this circumstance, the point of the director may be introduced into the rectum without lessening the chance of the success of the operation."

2d. I object to the dissecting out of the hardened integument at the edges of the fistula, which is done by two incisions crossing each other at right angles, the flaps being each lifted up with a pair of forceps, and cut out with the knife. What end is sought by this part of the operation? what object is there in view? what good can possibly result from it? what advantage attends this increase of suffering to the patient? The old surgeons supposed that there was something malignant in the hardness and callosity attending this disease, and were not content with opening the cavities, but endeavoured to dissect out the whole of the parts, and if unable to do this, they finished the work with the red-hot iron. Such practice may be worthy of a day gone by, but certainly not of the nineteenth century.

3d. I must beg leave to enter my most earnest protest against filling the wound daily with large quantities of charpie. This plan is attended with great pain, produces considerable uneasiness, and most certainly impedes the cure. The wound cannot be dressed too lightly, and the less it is disturbed the better. But if the surgeon crams it every day with lint or charpie, the sides of the cavity cannot contract; they become hard and callous, nor is there any thing in my opinion so likely to reproduce the disease as this system of filling it with dressings. Mr. Cooper, of University College, well remarks, "a sore so cramped will not permit the matter to escape. A patient, who has been so treated, has generally some degree of fever; has a pulse which is too hard and to quick; is thirsty; and does not get his due quantity of natural rest."

When a sore has been so treated, there always is a very considerable degree of inflammation about the verge of the anus, the lips of the wound are tumid and everted, red, and painful in a high degree, and all the lower portion of the bowel partakes of this state of things, producing frequent desire to go to stool, and considerable irritation; nor is any thing more calculated to produce that fatal erysipelas before described than this mismanagement of the wound. If we were called upon to treat such a case, the first thing would be to remove the dressings to foment the wound with warm water. At the same time, attention must be paid to the state of the system, and treatment directed to calm the general disturbance that may have arisen, made use of.

M. Roux, whose talents and great experience entitle any opinions he may express to the greatest possible attention and respect, in a critique, published some years ago, upon the manner of performing this operation in England, censures our plan of not filling the wound with charpie; but, says Mr. B. C. Brodie, "it is chiefly in consequence of the use of too much lint in dressing, that further operations are so frequently required before the cure is completed;" this opinion also coincides with the experience of Mr. Liston, who strongly reprobates the plan of filling the wound with lint or charpie.—*Dr. Hall, in Lond. Med. Gaz.*

---

*Method of detecting Poisoning by Copper.*—M. Orfila recently read a paper before the Academy of Medicine, in which he showed the method applicable to the detection of the salts of copper. We will content ourselves with translating his conclusions, as we find them in the Gazette Médicale of June 20, 1840.

It follows, he says, from the experiments and considerations detailed in his paper,—

1. That the acetate and sulphate of copper, when introduced into the stomach, or applied to the sub-cutaneous cellular tissue of living dogs, are absorbed, and carried into every organ in the system.

2. That it is probably the same in man.

3. That it is possible, by chemical means, to obtain metallic copper from the absorbed portion of the cupreous salts.

4. That it is indispensable to have recourse to this process, when these poisons have not been found in the alimentary canal, or in the other parts to which they had been immediately applied, or in the matter vomited; for if we limit ourselves, as has hitherto been done, to searching for the cupreous salts in the matters obtained from the stomach and intestines, we run the risk of not discovering them at all; either because none remains in the alimentary canal, or because the matters vomited have been removed; while the metal can always be obtained from the portion absorbed.

5. That a medico-legal report must in future be declared incomplete and insufficient, if, in the case just mentioned, the examiner has omitted to look for the cupreous salts in the parts where they are to be found after having been absorbed.

6. That besides the portion of the cupreous salt absorbed during life, and which is to be found unequally scattered over all the tissues, several organs (particularly the abdominal viscera, if the salts have been introduced into the alimentary canal,) contain some of the salt, which has reached them by imbibition after death. This happens more particularly on the part of their surface which is in contact with the alimentary canal; and the quantity varies according to the period when the body was opened. The copper obtained from these organs proceeds partly from what was absorbed during life, and partly from what had penetrated the tissues after death.

7. That the imbibition here mentioned, and which has been perfectly proved by the experiments of Fodera, Collard de Martigny, Magendie, Müller, &c., as well as by my own, is not peculiar to poisoning by copper, as it is observed whenever any poison, having been incompletely absorbed during life, remains upon the tissues after death; providing that the poison is dissolved, or is capable of being dissolved, in the liquid which touches it. And thus, what has been said of the proportion of the cupreous poison furnished by the viscera, whether in consequence of absorption or imbibition, is applicable to all cases in which the poisons have been absorbed.

8. That it is possible, in the majority of cases, to determine if the cupreous salts, or other poisons obtained from the viscera, in medico-legal investigations, have been introduced during life or after death, by paying attention to the symptoms which preceded death, and the structural lesions observed on opening the body, or by means of chemical experiments on some organ remote from the digestive tube, rather than on another which is near it, or on one part of a viscus rather than another. It is true, that in a few rare cases—as, for instance, if a long time has elapsed since the burial of the body, when nothing is left but the *detritus* of the viscera—the problem might be less easy to solve, unless the examination was assisted by the information collected by the magistrates, positively demonstrating that the poison was not introduced into the alimentary canal after death. The annals of justice, moreover, do not supply a single example of an accusation of poisoning, where wickedness has been carried to such a pitch as to introduce poison into the alimentary canal of a corpse, for the purpose of founding a charge.

9. That the absorbed cupreous salt, which caused the death of the patient, may be detected by boiling the viscera, or the flesh, for an hour in distilled water, drying the filtered decoction, and carbonizing it with nitric acid, or decomposing it by nitrate of potash.

10. That if boiling water does not dissolve the whole of the absorbed cupreous salt even in six hours, still enough is extracted to put its existence beyond the reach of doubt.

11. That boiling for an hour in distilled water does not dissolve any trace of the copper naturally existing in our frame; for this can only be partially separated by the concentrated acids, and totally by incineration alone. Hence the examiner may conclude, that some preparation of copper has really been introduced during life, either as a poison or as a medicine, if he obtains copper from an aqueous decoction prepared as above mentioned, unless it is proved that the copper penetrated the tissues in consequence of imbibition after death.

12. That it is best to boil, in the first place, the viscera which are distant from the alimentary canal; next, those portions of the abdominal viscera which have not been in contact with the canal; and then the parts which have been in contact with the stomach and intestines; for, by thus operating, we are certain to obtain from the latter a greater quantity of poison, and to collect information adapted to solve the questions which might be raised concerning imbibition.

13. That if the medico-legal researches, instead of relating to the viscera, referred to alimentary or excrementitious substances, contained either in the digestive canal or the liquids vomited, these substances must be boiled for an hour in distilled water, and the decoction is to be filtered, dried, and decomposed by pure nitric acid, or by nitrate of potash not containing any copper. The presence of copper, after the decomposition, enables us to affirm, that a cupreous preparation has been taken, unless, indeed, it has been introduced after death. Although boiling water dissolves only a small part of the salts of copper when they are intimately combined with organic matter, yet the solution contains enough of the metal to coat a plate of iron.

14. That if, after having treated these alimentary or excrementitious matters with boiling water, no copper is found, it would be wrong to treat them with the strong acids, or to incinerate them; because, even if copper should then be obtained from them, it would not prove that it had been taken as a poison or a medicine, since several alimentary substances contain enough copper to be detected by strong acids, and still more by incineration. It is better in such a case to give up the search for copper in these alimentary substances, and to treat the alimentary canal, the liver, spleen, kidnies, &c., with boiling water.

15. That while I admit, with M. Devergie, that the quantity of copper naturally contained in the intestines of an adult does not exceed 46 milligrammes, (9-10ths of a grain,) I cannot grant him that it is worth while, in forensic medicine, to take account of this copper, and decide, by incineration, whether the copper obtained is, or is not, that which is naturally present; because, as he says himself, the quantities of normal copper found in the few experiments he made, were too variable to permit him to consider the above-mentioned quantity as exact; and, still more, because it may easily happen that, after poisoning by a cupreous salt, so little remains in the intestines, that, after adding the copper thus introduced into them to that which they naturally contain, only 40 or 50 milligrammes can be obtained.—(See M. Devergie's *Médecine Légale*, 2d edit., vol. iii., p. 536-37.) The copper obtained by incineration can be taken into account only when it much exceeds the quantity which farther and multiplied experiments shall have shown to be the maximum of normal copper; but, even in this case, it is infinitely preferable to use the means I propose, because they furnish the clear and precise results, which I will conclude by repeating,—*Copper, which has been employed as a poison, may be obtained in part from viscera boiled in water for an hour; but not an atom of the copper naturally in them can be obtained in this manner.*

*Lond. Med. Gaz.*

---

*Death of Graefe.*—We regret to announce the decease of this distinguished surgeon, which took place on the fourth of the present month, (July,) at Hanover, whither he had repaired for the purpose of operating on Prince George of Cumberland, for cataract. His remains have been conveyed back to Berlin.

*Ibid.*

---

*Small-Pox Pustule in the Bladder.*—Dr. Greene presented a remarkable specimen of small-pox on the mucous membrane of the bladder. The patient, a young man, died of small pox. While convalescent from fever, he caught infection from another person in the same room, and had the disease in a confluent form. He died on the fifth day after the appearance of the eruption, and shortly before his death was attacked with severe diarrhœa. There were no pustules in the respiratory passages or intestinal tube.—*Dublin Journal of Medical Science.*

---

*Lumbrici in the Hepatic Ducts.*—Dr. J. Power exhibited the recent parts in this case; the ductus communis was distended with a large collection of lumbrici; its diameter exceeded half an inch; the hepatic duct was still larger, being in some parts more than an inch in diameter, and filled with lumbrici. The duct in the substance of the liver contained numbers of this description of worm, as did likewise the stomach and intestines. It was not known whether there were any symptoms of hepatic derangement during life, the specimen having been found in a subject brought into the anatomical room of the Richmond School of Medicine. (Museum, Richmond School.)

*Ibid.*

